# Neurobiological Mechanism of Acupuncture for Relieving Visceral Pain of Gastrointestinal Origin

**DOI:** 10.1155/2017/5687496

**Published:** 2017-01-24

**Authors:** Fang Zhang, Luyi Wu, Jimeng Zhao, Tingting Lv, Zhihai Hu, Zhijun Weng, Shuoshuo Wang, Huangan Wu, Huirong Liu

**Affiliations:** ^1^Shanghai Research Institute of Acupuncture and Meridian, Shanghai 200030, China; ^2^Shanghai TCM-Integrated Hospital, Shanghai University of Traditional Chinese Medicine, Shanghai 200082, China

## Abstract

It is currently accepted that the neural transduction pathways of gastrointestinal (GI) visceral pain include the peripheral and central pathways. Existing research on the neurological mechanism of electroacupuncture (EA) in the treatment of GI visceral pain has primarily been concerned with the regulation of relevant transduction pathways. The generation of pain involves a series of processes, including energy transduction of stimulatory signals in the sensory nerve endings (signal transduction), subsequent conduction in primary afferent nerve fibers of dorsal root ganglia, and transmission to spinal dorsal horn neurons, the ascending transmission of sensory signals in the central nervous system, and the processing of sensory signals in the cerebral cortex. Numerous peripheral neurotransmitters, neuropeptides, and cytokines participate in the analgesic process of EA in visceral pain. Although EA has excellent efficacy in the treatment of GI visceral pain, the pathogenesis of the disease and the analgesic mechanism of the treatment have not been elucidated. In recent years, research has examined the pathogenesis of GI visceral pain and its influencing factors and has explored the neural transduction pathways of this disease.

## 1. Introduction

According to the International Association for the Study of Pain (IASP), “pain is an unpleasant sensory and emotional experience associated with actual or potential tissue damage.” A thorough understanding of pain has not been clearly elucidated in the medical field. Research on somatic pain and neuropathic pain tends to be improved and perfect. However, the pathogenesis of visceral pain has not yet been clearly elucidated despite significant progress of relevant research. Visceral pain occurs in the interior organs (chest, abdomen, and pelvis) and is commonly observed in celiac diseases, such as gastrointestinal (GI) disorders, in the clinic. The manifestation of visceral pain is most typical in irritable bowel syndrome (IBS), and this disease is the most commonly used animal model in experimental studies of visceral pain. Visceral pain refers to pain from noxious stimuli such as painful swelling, ischemia, and inflammation that act on visceral organs via peripheral and central pathways [1]. There is a clear and unambiguous distinction between visceral pain and somatic and neuropathic pain. The characteristics of visceral pain include the following: (1) there is a vague sensation with an unclear position; (2) there is frequent accompaniment of referred pain in other areas such as the skin and muscle; (3) the generation of pain sensation is associated with motion and/or autonomic reflexes; and (4) persistent visceral pain can produce hyperalgesia in skin and deep tissues [2, 3]. Currently, the pathogenesis and influencing factors of GI visceral pain include visceral hypersensitivity, GI motility disorders, brain-gut axis abnormalities, intestinal infections, immune function changes, hereditary factors, and psychosocial factors.

Visceral pain is the most common symptom of functional bowel disorders and inflammatory bowel disease in the clinic. It is chronic in most cases and can be persistent or intermittent, which seriously affects the patient's life and work and costs substantial resources. Despite the excellent efficacy of EA in the treatment of GI visceral pain, the pathogenesis of this disease and the treatment mechanism of EA have not been investigated clearly. Over the past few years, research has examined the pathogenesis of GI visceral pain and its influencing factors and explored the neural transduction pathways of this disease. Visceral pain in IBS is a digestive disease commonly observed in clinical practice [4], but its etiology and pathogenesis have not been clarified. Epidemiological data from 2014 revealed that the worldwide prevalence rate of IBS was 3–22% [5]; this prevalence rate in China was 0.82–5.67% [6]. The clinical features of IBS cause significant inconvenience in the daily lives and work of patients and also reduce their quality of life. Therefore, the treatment methods and their efficacy for IBS are particularly critical. Modern medical treatment of GI visceral pain primarily consists of medication therapy. Despite its efficacy, long-term medication use is associated with side effects. Traditional Chinese medicine, particularly acupuncture and moxibustion therapy, has a long history and has demonstrated significant effects in pain treatment. Because it has good long-term effects without toxic side effects or recurrence, EA has been approved and recommended by the World Health Organization (WHO) as the main method of pain relief [7, 8]. According to existing studies, the possible analgesic mechanisms of EA treatment for GI visceral pain can be summarized in two aspects: the peripheral and central nervous pathways.

**Figure 1:**
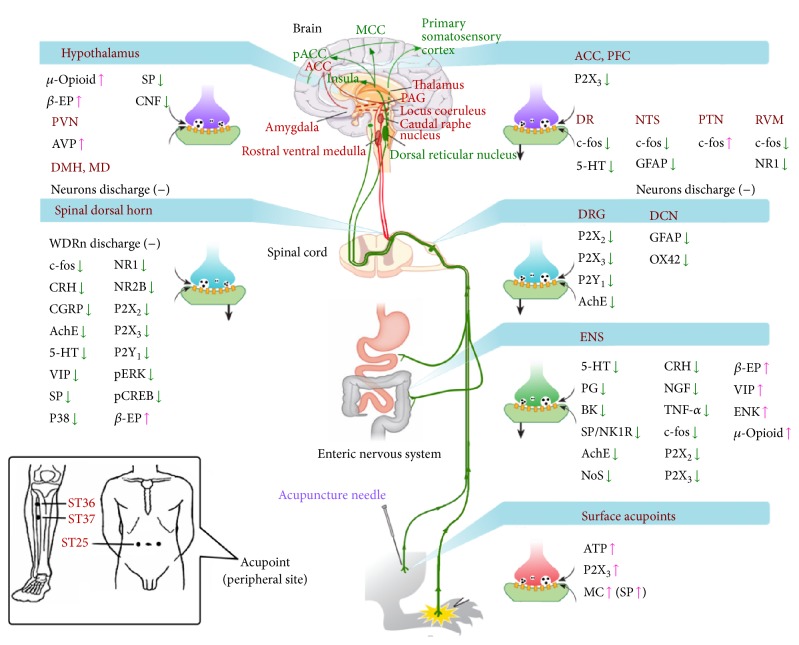
Ascending and descending pathways of the endogenous pain modulation system mediating visceral pain sensation in the brain-gut axis. Picture quoted from Zhang et al., Anesthesiology (2014) [11] and Moloney et al., Front Psychiatry (2015) [17]. RVM: rostral ventromedial medulla; MD: mediodorsal thalamic nucleus; PFC: prefrontal cortex; ACC: anterior cingulate cortex; DR: dorsal raphe nucleus; DMH: dorsomedial hypothalamic nucleus; AVP: arginine vasopressin; CNF: corticotropin releasing factor; GFAP: glial fibrillary acidic protein; WDR: wide dynamic range neurons; BK: bradykinin; PG: prostaglandins; *β*-EP: *β*-endorphin; (−): inhibition of cell discharge; NTS: nucleus of the solitary tract; TNF-*α*: tumor necrosis factor-*α*; NK1R: neuropeptide K 1R; SP: substance P; ENK: enkephalin.

The neurobiological mechanism is one of the most important analgesic mechanisms of acupuncture [9]. EA stimulation of a surface acupoint activates the enteric nervous system (ENS), leading to the release of varying levels of neurochemical signaling molecules from the brain-gut axis, such as 5-HT, norepinephrine, bradykinin, histamine, and encephalin [10, 11] (Figure 1). Furthermore, these molecules inhibit inflammatory reactions or promote damage repair, interfere with the afferent peripheral sensory nerve impulses, and break the vicious noxious stimuli-pain cycle, eventually relieving the pain. In recent years, numerous scholars have made significant progress in research and discussion with respect to the mechanism of EA treatment for GI visceral pain. This paper summarizes the recent knowledge on the neurobiological mechanism of EA for relieving GI visceral pain and further discusses the new advances and directions in EA treatment of visceral pain.

## 2. Transduction Pathways of GI Visceral Pain

It has been accepted that the neural transduction pathways of GI visceral pain are divided into peripheral and central pathways. Existing research on the neurological mechanism of EA treatment for GI visceral pain has primarily focused on the regulation of these transduction pathways. (1) The peripheral pathway: noxious stimuli act on the receptors in the GI mucosa and are transmitted from primary afferent nerve fibers to primary sensory neurons in the dorsal root ganglia (DRG). Primary afferent nerve fibers include extrinsic and intrinsic afferent fibers that govern the GI tract. Extrinsic nerves refer to sympathetic and parasympathetic nerves, and sympathetic afferent fibers transmit signals to the spinal DRG. Parasympathetic afferent fibers include two pathways through the vagus and pelvic nerves, respectively. The afferent information from the vagus nerve is mainly relayed through the nucleus tractus solitarius (NTS) in the central nerves; the afferent information from the pelvic organs is mainly relayed through the sacral dorsal commissural nucleus (DCN) in the central nerves. Intrinsic nerves refer to the ENS, which mainly includes the submucosal plexus and the myenteric plexus. These nerves belong to vagal afferent fibers that directly transmit signals into the NTS of the medulla [12, 13] (Figure 1). (2) The central pathway: (1) information is transduced in the primary sensory neurons of the spinal DRG and then transmitted to the spinal dorsal horn neurons (DHN), wherein nociceptive information is subject to primary central integration, followed by ascending transmission in the spinal cord through the spinothalamic tract, the spinoreticular tract, and the spinomesencephalic tract to the thalamus, the reticular formation, and the midbrain; further, the information is projected to the cerebral somatosensory cortex, the anterior cingulate cortex (ACC), and the insular cortex, resulting in visceral pain [14]. (2) NTS conduction of information: more afferent information is transmitted from the visceral portion of the NTS (caudal medial part) to the nucleus parabrachialis and then to the ventral posterior nucleus and the parvicellular part in the thalamus, finally reaching the insular cortex and constituting the main visceral afferent central pathway; part of the afferent information is transmitted along the NTS to the DCN and then to the hypothalamus and amygdala pathways, which are primarily responsible for emotional changes because of visceral sensation [15]. (3) The cingulate gyrus, amygdala, midbrain periaqueductal gray matter, and rostral ventromedial medulla (RVM) constitute the descending pathway; central signals from the nerve fibers of the RVM are projected through the ventrolateral funiculus and dorsal lateral funiculus to the spinal cord and are mainly terminated in the spinal DHN [16] (Figure 1).

## 3. Neurological Mechanisms of EA for Relieving GI Visceral Pain

EA analgesia has been applied in traditional Chinese medicine for thousands of years. It is now extensively used in clinical practice [18], and increasing attention has been received from clinicians and researchers in many countries. The range of treatment includes pain and inflammatory diseases. Body surface stimulation can suppress painful feelings in visceral pain. In traditional Chinese medicine, AP, moxibustion, and massage achieve this purpose by stimulating surface acupoints. These phenomena involve complex neurobiological mechanisms. It is difficult to explain them by the traditional lower center convergence-projection theory of referred pain and the classical pain transduction pathway. EA therapy and other surface physical stimulation therapies in modern medicine (e.g., lumbosacral magnetic stimulation, transcranial magnetic stimulation, and transcutaneous electrical nerve stimulation) have been applied and have been widely used in the clinic worldwide; these methods are safe and effective [18, 19]. Since the 1980s, the neurological mechanism of EA treatment for GI visceral pain began to receive sustained attention worldwide. Although the neurological mechanism of EA for relieving GI visceral pain has been partially revealed, specific transduction pathways in EA treatment have not been found and must be determined in further study (Figure 1).

### 3.1. Peripheral Neurological Mechanism of EA for Relieving GI Visceral Pain

Recent studies show that visceral hypersensitivity is one of the main pathophysiological bases of GI visceral pain. As described earlier, the pathogenesis of GI visceral pain includes the peripheral and central mechanisms. Previous research on the peripheral mechanism has primarily been concerned with how noxious stimulation acts on the receptors in the GI mucosa and activates mast cells to secrete various inflammatory mediators, such as 5-HT, prostaglandins, and bradykinin; these factors act on the corresponding receptors on the sensory nerve endings [20], thus transmitting nociceptive information to the spinal dorsal root. Research on the peripheral neurological mechanism of EA for relieving GI visceral pain has primarily focused on neurons and associated neurotransmitters and afferent fibers.

#### 3.1.1. Enteric Nervous System (ENS)

Over the past few years, numerous studies have found that the brain-gut axis plays a critical role in the development and progression of visceral pain. The brain-gut axis refers to the physiological and pathological phenomenon in which the central nervous system (CNS) and the GI tract mutually affect and regulate one another through neurotransmitters and chemical or electrical signals. It has a role in various functionality-, motility-, and immune-related GI disorders [21] (Figure 1). Meanwhile, the brain-gut axis forms the physiological basis of AP for regulating the GI function. The peripheral pathway of GI visceral pain mainly refers to the primary sensory neuron stage of signal transmission through primary afferent nerve fibers to the spinal DRG after noxious stimulation acting on the receptors in the GI mucosa. Presently, it is widely accepted that noxious visceral afferent nerve fibers comprise thin myelinated A*δ* fibers (30–40%) and unmyelinated C fibers (60–70%) [12, 22]. During pain treatment with AP, needling sensation is a prerequisite for an analgesic effect. Electrophysiological studies found that needling sensation impulses are mainly governed by A*δ* fibers (class III) and C fibers (class IV), which transmit the needling sensation to the upper spinal center, thus achieving the analgesic effect [23]. However, once AP excites afferent nerve fibers deep in the acupoint area, the pain threshold value will be increased. At the same level of nerve segment, AP in the Hegu acupoint can increase the pain threshold value deep in the acupoint itself. Such AP can achieve an obvious analgesic effect as long as it excites class II and a small amount of class III fibers deep in the acupoint area. For the distal segment, the AP analgesic effect requires the participation of C fibers. From these studies [24–26], it can be shown that the convergence of AP signals and visceral noxious afferent neurons in the spinal cord and the upper center is the neurobiological basis of AP for relieving visceral pain. AP can produce certain analgesic effects as long as it activates A*δ* (class III) or C (class IV) fibers.

In the peripheral pathway of GI visceral pain, the ENS functions independently of the CNS and is also known as the “gut cerebellum.” In the ENS, cholinergic neurons of the submucosal nervous plexus and the intestinal myenteric plexus can release an important neurotransmitter called acetylcholine (AchE). AchE is considered to be the primary neurotransmitter used to regulate GI motility [27] and to participate in the primary afferents of analgesic information of AP [28]. Early animal studies found that cholinergic nerves are involved in the transmission of noxious visceral pain sensation in the rat intestinal tract with acute inflammation. Electroacupuncture (EA) can reduce the AchE that has increased during inflammatory reactions to relieve visceral pain [29]. The EA stimulation (50 Hz) of rats with IBS visceral pain at acupoints ST25 and ST36 significantly reduced the visceral hypersensitivity that was induced by mechanical colorectal distension (CRD). Meanwhile, it was found that EA could downregulate the amount of mast cells, SP, vasoactive intestinal polypeptides (VIPs), neurokinin-1 receptors, and VIP receptors [30, 31] and CRH [32], NGF, and NGFR expression [33] in the descending part of the colon in the target organ, making the AWR score lower.

5-HT, as a brain-gut peptide, is widely present in the CNS and GI tract and functions as an important neurotransmitter to regulate functions of the digestive tract [34]. 5-HT_3_ receptors are massively distributed in the myenteric nerve plexus (primary afferent neurons) and participate in the regulation of abdominal discomfort symptoms in IBS VP [35, 36]. Clinical studies showed that 5-HT and 5-HT_3_ expression levels were markedly increased in the intestinal mucosa among patients with IBS; the application of a 5-HT3 receptor antagonist improved the threshold value of colonic CRD [37, 38]. EA stipulation of acupoint ST36 at 100 Hz downregulated colonic levels of 5-HT and 5-HT_3_ expression in the brain-gut axis [39–41], further reducing pain symptoms. Moreover, it was found that EA could regulate serum levels of 5-HT in patients with visceral hyperalgesia in clinical treatment. SP, 5-HT, and histamine released from mast cells mediated the sensitization of visceral afferent fibers [42]. Patients with irregular abdominal pain exhibited higher expression levels of colonic VIP, NK1R, and TNF-*α* mRNA than normal controls [43, 44]. Thus, it appears that EA downregulates peripheral chemicals to reduce the sensitivity of the splanchnic nerves, thereby achieving the effect of relieving visceral pain. However, there are no experimental designs available concerning specific antagonists and gene knockouts. Moreover, animal studies revealed that 20-Hz prestimulation of acupoint Jiaji markedly reduced the behavior response of visceral pain hyperalgesia as induced by the intestinal injection of formalin. Meanwhile, it reduced the phosphorylation of colonic mucosa P38 and downregulated fos expression but upregulated *β*-endorphin expression. Such effects were not observed in normal rats [45].

In the body, the epithelial cells of tubular and saclike organs (e.g., the intestines, ureter, and bladder) release ATP upon mechanical distention stimulation. P2X3 and P2X2/3 receptors act on the submucous nerve plexus in the epithelium and induce pain signals to be transmitted towards the center [46, 47]. P2X receptors (particularly the P2X2 and P2X3 subtypes) participate in the conduction and modulation of visceral nociceptive information in the peripheral and central nervous systems and are highly selectively expressed in sensory neurons [48]. Behavior, morphology, and molecular biology experiments revealed that P2X3 receptors mediate the pathogenesis of visceral pain in peripheral and central neurons in IBS rats. Fibers of P2X3 receptor-positive immunoreactive neurons are projected to spinal dorsal horn (SDH) II with the expression in these afferent nerve endings [49] being significantly positively regulated by EA [50, 51].

### 3.2. Spinal Neurobiological Mechanism of EA for Relieving GI Visceral Pain

The main pathogenetic mechanism of visceral pain is visceral hypersensitivity, and the pathogenesis of visceral hypersensitivity includes the peripheral and central mechanisms. However, central sensitization has been found to be a key factor in the development and progression of visceral hypersensitivity. If central sensitization is inhibited, then chronic visceral pain can be effectively relieved [52] (Figure 1). Central sensitization is a complex process that involves various neurons, nerve nuclei or nuclei, and neurotransmitters involved in nerves. In research on the central nervous mechanism of EA for alleviating GI visceral pain, part of the mechanism was identified by functional magnetic resonance imaging [53], which may be consistent with its central conduction pathway. However, further research is still required to fully reveal the central mechanism of EA analgesia.

#### 3.2.1. Dorsal Root Ganglion (DRG)

In the central transduction pathway of visceral pain, the spinal cord is the first level in the integration center of pain signals after entry to the central nerves. It is the relay station of afferent pain information, and it directly modulates the pain sensation and also receives the descending regulatory signals of the upper spinal center; thus, the spinal cord is regarded as a key part in the regulation of pain stimulation [54]. The DRG, as the first level of neurons for afferent sensory information, has an extremely important role in transmitting information between peripheral and central nerves [55]. During the treatment of visceral pain, EA may inhibit the excited DRG neurons and the expression of related neurotransmitters and receptors through afferent impulses at the acupoint, thereby achieving an analgesic effect. The majority of experimental studies in rats with visceral hyperalgesia have found that the model rats presented with significant hyperalgesia to CRD, with markedly higher excitability of DRG neurons. EA stimulation of acupoint Zusanli was able to reduce the excitability of the DRG neurons and the expression of related neurotransmitters and receptors, thus remarkably relieving the symptoms of visceral hyperalgesia [56, 57]. This effect of EA was blocked by an intraperitoneal injection of naloxone. Moreover, DRG neurons can convey sensory impulses from the peripheral to the central nerves and then to the SDH, completing the transmission of primary sensory information. It was postulated that, after reducing the excitability of DRG neurons, AP may block the pathway of pain signals to a higher degree. Moreover, P2X3 receptors of DRG sensory neurons have an important role in ATP-mediated pain in IBS rats with visceral hypersensitivity [58]. The upregulated expression of P2X3 receptors was implicated in the DRG neurons of visceral pain model rats prepared by CRD, and EA was able to reduce the expression of P2X3 receptors in DRG cells [50]. The role of P2X3 receptors in the activation of nociceptors in IBS rats was further explored at the gene level by a real-time PCR analysis. Experimental evidence demonstrated that EA could relieve visceral hyperalgesia in rats with visceral pain by reducing the expression of P2X2 and P2X3 receptors in the colon and spinal cord [59] and P2Y1 receptors in DRG cells. Thus, the spinal cord and upper spinal centers have a critical role in the EA treatment of visceral pain.

#### 3.2.2. Spinal Dorsal Horn (SDH)

The SDH plays an important role in the transmission and regulation of visceral nociceptive information. It converges visceral afferent nerves from the periphery, descending projection nerves from the senior center, and SDH neurons, thus forming a complex neural network. The SDH contains abundant neurotransmitters and associated receptors, neuromodulators, and ion channels, which not only receive and transmit nociceptive information but also preliminarily process nociceptive information [54, 60, 61]. Research of the visceral pain model in rats has found that CRD can activate the response of wide dynamic range (WDR) SDH neurons, whereas the AP stimulation of acupoints can inhibit the neuronal response activated by visceral nociceptive afferents and thus alleviate visceral pain [62]. However, SDH neurons participate in the descending transduction pathway of visceral pain. The information of AP can be conveyed through SDH neurons to the upper spinal center, thus activating the descending regulation system of pain; this mechanism contributes to the expression of SDH receptors, such as 5-HT, and further achieves an analgesic effect [63]. As stated earlier, the EA prestimulation (20 Hz) of acupoint Jiaji markedly reduced the phosphorylation of P38, downregulated fos expression, and upregulated *β*-endorphin expression in colonic mucosa; a similar effect of EA was found in the SDH [45]. As described, EA can inhibit the release of algogenic neurotransmitters by regulating the activity of endogenous opioid peptides in the spinal cord and the DRG, thereby achieving the purpose of alleviating visceral pain [64]. The spinal cord of the central level has provided an important neurobiological basis for the effect of EA in alleviating visceral pain. Meanwhile, it has a critical role in regulating inflammatory and pathological pain. Recent studies have found that the EA stimulation of acupoint Shangjuxu significantly reduced visceral hypersensitivity and lowered the pain threshold in rats with IBS induced by CRD; moreover, CRH and its mRNA expression appeared abnormal in the peripheral target organs, colon, and spinal cord [32]. Thus, EA stimulation of acupoint Shangjuxu also achieves a therapeutic effect through this pathway in IBS rats with visceral hypersensitivity.

The spinal cord is the first level of the integration center for pain signals that enter the central nerves. It directly modulates pain sensation and simultaneously receives descending regulatory signals from the upper spinal center; thus, it also regulates the transmission of visceral nociceptive information. AP signals are conveyed through SDH neurons to reach the upper spinal center; once the descending modulation system of pain is activated, AP regulates the expression of SDH c-fos, p38, and 5-HT receptors to achieve an analgesic effect [65, 66]. Additionally, EA can markedly inhibit the expression of c-fos and NMDA-R1 receptors in the L6–S2 segments of the SDH and RVM [67–70] and can reduce the abnormally high excitability of visceral response neurons in the SDH and RVM. As described, the regulation of visceral pain sensation in IBS rats is achieved through multiple receptors at the spinal cord level. Experimental data have shown that EA can alleviate visceral hyperalgesia by reducing c-fos, P2X2, and P2X3 receptor expression in the colon and spinal cord [59] and P2Y1 receptor expression in the DRG cells of rats with visceral pain. Thus, the spinal cord and the upper spinal center play an important role in the analgesic mechanism of EA for visceral pain [71, 72].

#### 3.2.3. Sacral Dorsal Commissural Nucleus (DCN)

The DCN is located in the dorsal central canal of the sacral spinal cord. It is the projection site of primary afferent signals from the pelvic organs. The DCN participates in the transmission and regulation of pain signals in the left semicolon and acts as a relay station of the brain-gut axis in the pain pathways. DCN neurons have been shown to be activated by the colonic inflammation-induced visceral pain response, and the activated neurons in turn can preliminarily integrate noxious stimuli from the colon, thereby regulating the visceral pain response [73–75]. Moreover, recent studies have shown that glial cells play an important role in pain [76]. EA stimulation of acupoint Zusanli significantly attenuated the visceral pain response induced by noxious stimulation and inhibited DCN, glial fibrillary acidic protein (GFAP), and OX42 expression, indicating that glial cells (astrocytes and microglia) in the DCN participate in the analgesic process of EA [77]; the activation time of astrocytes is earlier than that of microglia [78]. Meanwhile, there is the “exchange of information” and “conversation” between glial cells and neurons, with mutual “activation” between one another [79]. It was further postulated that AP has an inhibitory effect on the activated glial cells in the DCN of rats with visceral pain, thereby posing an inhibitory effect on the excited neurons in the DCN and further playing an analgesic effect or blocking the transmission of pain information to the upper center. This postulation must be verified in further studies.

### 3.3. Upper Spinal Neurobiological Mechanism of EA for Relieving GI Visceral Pain

#### 3.3.1. Thalamic and Brainstem Reticular Neurons

The thalamus and brainstem reticular formation are important centers for processing pain information and integrating pain sensation. Thalamic and brainstem reticular neurons are the third level of pain information transmission. AP can influence nociceptive information transmission of the thalamus and brainstem reticular formation in the upper spinal center [53, 80, 81] (Figure 1). Neurophysiological research shows that neurons that respond to noxious stimuli exist at various levels of the CNS [82]. Early studies found that hypothalamic vasopressin neurons and the paraventricular nucleus participate in the inhibitory mechanism of EA for visceral pain induced by the intraperitoneal injection of antimony potassium tartrate [83]. The EA stimulation of acupoints ST36 and ST37 EA markedly relieved visceral pain and simultaneously upregulated hypothalamus *β*-endorphin and SP expression [84] and CRF synthesis [32, 85] in a rat model of visceral pain induced by mechanical distension of the stomach and colon. Moreover, EA stimulation of acupoint Zusanli inhibited visceral pain; fos expression in the brainstem nucleus raphes dorsalis, shallow SDH, and colonic epithelium; and 5-HT expression in the nucleus raphes dorsalis and the SDH in a rat model of visceral pain caused by neonate-mother separation [63]. This finding suggests that the thalamic mediodorsal nucleus (MD) is involved in information transmission of not only visceral pain but also AP. EA stimulation of acupoint Zusanli markedly inhibited the discharges evoked by pain-excited neurons of the thalamic MD but increased the discharges of the pain-inhibition unit in a rat model of visceral pain, thus producing an analgesic effect [86]. Additionally, visceral pain afferent signals can cause discharge reactions in thalamic and brainstem reticular neurons and thus cause the possible convergence of two sensory afferent signals (impulses from the AP site and visceral pain) in these thalamic and brainstem reticular neurons. AP can inhibit discharge reactions in visceral pain through certain integration mechanisms of the center, thereby alleviating visceral pain [81]. The stimulation at “Zusanli-Shangjuxu” acupoints enhanced discharge activity of VPL neurons under CRD-induced visceral pain. The frequency of neuronal discharge was associated with the pressure gradient of CRD which showed that visceral noxious stimulation may intensify the body's functional response to stimulation at acupoints [87].

The electronic stimulation of skin receptive fields and acupoint Zusanli can inhibit the response of somatic and visceral convergence neurons of the thalamic ventrobasal nucleus to the CRD [88]. The skin receptive fields of thalamic neurons that are responsive to visceral nociceptive sensations are mainly located on the stomach meridian in traditional Chinese medicine. Therefore, stimulation of the receptive fields will generate a stronger inhibitory effect compared to acupoint Zusanli.

#### 3.3.2. Nucleus Tractus Solitarius (NTS)

The NTS is located in the dorsal medial part of the medulla oblongata. It acts as the relay nuclei of the visceral primary afferent fibers, which receive afferent information from the peripheral nerves and the spinal cord or medulla oblongata. Meanwhile, the NTS participates in the transmission of visceral nociceptive pain information and is an important central passageway for nociceptive afferent fibers and integration and regulation of visceral pain sensation [89]. The c-fos serves as a marker of active neurons [90], and the expression of glial fibrillary acidic protein (GFAP) is a sign of active glial cells [76]. Both c-fos and GFAP participate in the regulation of GI visceral pain. Research on the mechanism of EA for relieving GI visceral pain found that AP pretreatment markedly reduced c-fos positive neurons and GFAP expression in the NTS in model rats with GI visceral pain; these results suggest that the regulatory process of AP in visceral pain is closely related to the NTS [91]. Meanwhile, CRD can induce an excitatory response in related neurons in the NTS whereas EA stimulation poses an inhibitory effect on these neurons; these findings provide electrophysiological evidence that the NTS receives the afferent information of CRD-induced visceral pain and participates in the analgesic process of AP [92, 93] (Figure 1).

#### 3.3.3. Rostral Ventromedial Medulla (RVM)

The RVM is located at the central junction of the pontine reticular formation. It plays a critical role in the central regulation of pain and additionally serves as a common pathway of the upper spinal center for descending regulation of visceral noxious stimuli. The RVM has a dual role in regulating visceral pain, and it may inhibit or facilitate the input of noxious stimuli [94, 95]. The RVM can induce analgesia when receiving high intensity electrical stimulation and high concentrations of certain excitatory neurotransmitter microinjections; however, it will promote the pain response when receiving low intensity electrical stimulation or low concentrations of certain excitatory neurotransmitters [54]. Experimental studies found that, after CRD stimulation, model rats presented with increased excitability of visceral responsive neurons in the RVM, with an abnormal increase in c-fos positive neurons; EA treatment markedly inhibited the expression of c-fos positive neurons in the RVM of model rats with IBS and thus reduced the abnormally high excitability of visceral responsive neurons in the RVM. This may be one mechanism by which AP alleviates chronic visceral hyperalgesia. EA stimulation (5–100 Hz) of acupoint Zusanli can reduce the AWR score and IBS-induced glutamate N1 and fos overexpression in the RVM [72]. Application of the NMDAR antagonist in the RVM could inhibit visceral pain [70, 96], indicating that EA inhibits the activation of NMDA in the RVM to alleviate visceral pain.

#### 3.3.4. ACC and Prefrontal Cortex (PFC)

Nerve nuclei such as the PFC, ACC, thalamic medial nuclei, amygdala, midbrain periaqueductal gray matter, and caudate nucleus that participate in pain sensation and modulation have extensive fiber links and are important centers of pain sensation [97–99]. After being transmitted through the ascending pathways such as the pelvic and splanchnic nerves, visceral pain is integrated in the ACC. PET revealed the activation of the ACC in IBS patients after CRD [100]. In situ hybridization and immunohistochemical studies indicated that P2X3 receptors are expressed at certain levels in the PFC and ACC of adult rats; mechanical CRD could upregulate P2X3 expression in the PFC and ACC; moreover, EA stimulation of acupoint Shangjuxu could regulate the P2X3 receptors in the PFC and ACC [57], with an excellent modulatory effect on the degree of central sensitization and visceral hyperalgesia.

## 4. Others

Under the rectal balloon distension plus electroacupuncture condition, stimulation by electroacupuncture at Tianshu (ST 25) manifested a decreased regional cerebral metabolic rate of glucose in the left cingulate gyrus, right insula, right caudate nucleus, fusiform gyrus, and hippocampal gyrus. Electroacupuncture therapy relieved abdominal pain, distension, or discomfort by decreasing glucose metabolism in the brain [101]. A few studies recently published in Nature Medicine have shown that EA excites vagus nerves by surface stimulation of acupoint Zusanli, further leading to a strong systemic anti-inflammatory effect [102, 103]. This anti-inflammatory effect is achieved through dopamine, and its pathophysiological process typically reflects the function of the nerve-endocrine-immune network. It remains unclear whether this anti-inflammatory effect plays a role in the mechanism of EA for relieving inflammatory visceral pain (e.g., IBD). Nonetheless, all of these findings provide a good basis for revealing the mechanism of EA treatment for inflammatory visceral pain.

## 5. Conclusion and Outlook

Under physiological and pathological conditions, the processing of pain information by the body generally has cross-level features. Although research has been conducted on both the cellular and the molecular levels (e.g., chemical transmission mechanism of signals), there is also a higher level of exploration including serial and parallel processing of signals that eventually form sensory perception. With the continuous development of science and technology, research on EA analgesia has entered the molecular neurobiology level. This paper systematically discusses the major neurobiological mechanisms of EA analgesia for GI visceral pain from the aspects of the peripheral and central pathways. Various pathways and substances are involved in the mechanism of EA for alleviating visceral pain. Although both peripheral and central nerves participate in the analgesic mechanism, most of our information has been derived from elucidation of in vitro animal experiments. There remains a lack of verification by in vivo functional experiments and clinical trials. Further clarification is necessary to understand how these structures jointly function during the analgesic process of EA. Additionally, most existing studies have observed the regulatory effect of EA on neurotransmitters at the peripheral and central levels to discuss the possible mechanism of EA analgesia. However, relatively accurate neural pathways of EA analgesia have not yet been revealed because of the high complexity and diversity of nerve distribution in the transmission and modulation of visceral pain. Meanwhile, experimental research on EA for alleviating visceral pain is often focused on functional GI disorders, particularly IBS, as a model to investigate the underlying mechanism. Thus far, research has been lacking on AP analgesia of visceral pain in inflammatory GI disorders. Therefore, continuous investigation of the neural pathways of EA analgesia will be the priority of future research.
